# An Introductory Lecture, Delivered in the Anatomical Theatre of the University of Maryland, on the 1st November, 1836

**Published:** 1837-07

**Authors:** 


Art. III.—1. *An Introductory Lecture, delivered in the Anatomical Theatre of the University of Maryland, on the 1st November,* 1836. By Robert Eglesfeld Griffith, m.d., Professor of Materia Medica, Therapeutics, Hygiene, and Medical Jurisprudence.—*Baltimore,* 1836. 8vo. pp. 14.

2. *An Inaugural Address on Medical Eclectism.* By James Conquest Cross, m.d., Professor of Materia Medica in the Medical College of Ohio, &c.—*Cincinnati,* 1835. 8vo. pp. 20.

Professors of materia medica have the usage, if not the right, of lecturing upon all manner of subjects; and of being rhetorical, and even poetical in their public discourses. When the graver themes of cinchona and hydrargyrum fail them, they can dilate movingly upon errhines, and be affecting upon narcotics; lively upon opium, and smart upon snuffs. We have seen the lecture-table of one of our most distinguished lecturers on materia medica covered with skulls, to illustrate the physical history of mankind; and the operation of favorite medicines is, we believe, sometimes illustrated by a prodigious slaughter of the lower animals before admiring classes assembled to learn posology.

The two American professors of whose lectures we have given the titles, seem actuated by even a wider ambition, and have so astounded their hearers by their eloquence, that each lecture is published at the request of students affected with stupendous amazement. Both lectures are on general subjects, and in neither are a dozen consecutive lines to be found on the subject of materia medica, properly so called. Both are printed in a type somewhat difficult to read, and both on very indifferent paper.

It is really to be regretted that Professor Griffith, in whose lecture we see many opinions in which we fully concur, should have expressed himself in general so grandiloquently. After recommending, for instance, in unexceptionable terms, the combination of general information with professional acquirement, he adds, quite unnecessarily for the enforcement of his argument, the following lines:

> "The flimsy garniture required for the mere business of life, like the net of the retiarius, can only be employed in the attack, whilst the substantial panoply that qualifies its wearer for every emergency, like the arms of the soldier, give a form and an energy to the limbs that command respect and ensure success." (P. 8.)

Professor Cross is a still greater offender; but there may be something peculiar in the far-west, to us unknown, which would account for the peculiarities of his address.

> "Your profession," he says to the gaping and ingenuous back-woodsmen of tender age, "is in the hands of the Philistines; they have crushed its energies; they have blasted its prospects; they have covered it with disgrace; and it is now overspread by the dark and dreary night of desolation. Pause not till you have rescued it from their unhallowed keeping; rest not till you have accomplished its regeneration; sleep not till you have redeemed it from under the yoke of ignominious bondage!"

We think we see the breaking-up of a class thus addressed. The zeal of youth is extreme, and the consequences to the windows of shops are not to be calculated upon, excepting that, by the principle of *Eclectism*, some peaceful citizens may escape from the wrath of these regenerators, thus told to arise or be for ever fallen.

"We have been cut off," he continues, "from intercourse with the profession abroad. We have been sent into exile, and there has been erected in the far-west a medical empire, based upon principles so novel as to be without a parallel on earth; principles, which in their practical bearings, lead to consequences so disastrous in their character, that he who can look upon them without feeling his bosom bursting with the most painful emotions, must be dead to the voice of censure and callous to the accents of applause." And after much more to the same purpose, setting forth how the very children look upon a Western doctor "as a monster in human shape, waving aloft in stupid triumph the terrific banner of Azræl," he concludes his exhortation thus—

> "Does a family emigrate to our country? They are told to eschew a physician who has been educated in the west as they would the pestiferous effluvia of a charnel house. Shall this be? Shall the phrase '*Western physician*,' still continue the mortifying synonym of '*licensed murderer?*' Must this complimentary cognomen still remain attached to your names? No, I cannot, I will not believe it. You will rise in the majesty of strength—boldly breast the torrent, and roll back to its source the dark deluging flood of desolation."

We could quote many passages of equal fervour; but we have no wish to do so. Dr. Cross has been educated in Paris, and we doubt not that his talents and zeal, notwithstanding the superabundance of ornament with which his thoughts lie overwhelmed, will rouse the far-west men, and give them a better name.

